# Increased Circulating Malondialdehyde-Modified Low-Density Lipoprotein Level Is Associated with High-Risk Plaque in Coronary Computed Tomography Angiography in Patients Receiving Statin Therapy

**DOI:** 10.3390/jcm10071480

**Published:** 2021-04-02

**Authors:** Keishi Ichikawa, Toru Miyoshi, Kazuhiro Osawa, Takashi Miki, Hiroshi Ito

**Affiliations:** 1 Department of Cardiovascular Medicine, Okayama University Graduate School of Medicine, Dentistry and Pharmaceutical Sciences, Okayama 700-8558, Japan; ichikawa1987@gmail.com (K.I.); tm.f20c.2000@gmail.com (T.M.); itomd@md.okayama-u.ac.jp (H.I.); 2 Department of Cardiovascular Medicine, Japanese Red Cross Okayama Hospital, Okayama 700-8607, Japan; rohiwasa@yahoo.co.jp

**Keywords:** malondialdehyde low-density lipoprotein, high-risk plaque, coronary computed tomography angiography, statin

## Abstract

Objective: To evaluate the association of serum malondialdehyde low-density lipoprotein (MDA-LDL), an oxidatively modified LDL, with the prevalence of high-risk plaques (HRP) determined with coronary computed tomography angiography (CTA) in statin-treated patients. Methods: This study was a single-center retrospective cohort comprising 268 patients (mean age 67 years, 58% men) with statin therapy and who underwent coronary CTA for suspected stable coronary artery disease. Patients were classified into two groups according to median MDA-LDL level or median LDL-C level. Coronary CTA-verified HRP was defined when two or more characteristics, including positive remodeling, low-density plaques, and spotty calcification, were present. Results: Patients with HRP had higher MDA-LDL (*p* = 0.011), but not LDL-C (*p* = 0.867) than those without HRP. High MDA-LDL was independently associated with HRP (odds ratio 1.883, 95% confidential interval 1.082–3.279) after adjustment for traditional risk factors. Regarding incremental value of MDA-LDL for predicting CTA-verified HRP, addition of serum MDA-LDL levels to the baseline model significantly increased global chi-square score from 26.1 to 32.8 (*p* = 0.010). Conclusions: A high serum MDA-LDL level is an independent predictor of CTA-verified HRP, which can lead to cardiovascular events in statin-treated patients.

## 1. Introduction

Cardiovascular disease is the leading global cause of adult mortality and morbidity [1]. Many studies have demonstrated a beneficial effect of low-density lipoprotein (LDL)-lowering therapies by statins on cardiovascular outcomes [2,3]. Lowering low-density lipoprotein cholesterol (LDL-C) is the primary target in cardiovascular disease prevention; however, despite effective LDL-lowering treatment, substantial patients remain at cardiovascular risk [4]. Among several lipid markers, serum malondialdehyde low-density lipoprotein (MDA-LDL), which is an oxidatively modified LDL, has been reported to be associated with coronary plaque vulnerability [5], adverse clinical outcomes after percutaneous coronary intervention [6], and the incidence of acute coronary syndrome [7]. However, the clinical relevance of serum MDA-LDL levels in patients receiving statin therapy for cardiovascular events has not been fully elucidated.

Coronary computed tomography angiography (CTA) is used to noninvasively evaluate coronary artery disease [8]. In addition to the evaluation of stenosis, coronary CTA identifies characteristics of plaque composition. Many observational follow-up studies have demonstrated the association between high-risk plaques (HRP) by coronary CTA and cardiovascular events [9,10]. Although statins contribute to the stabilization of plaques [11], HRP is often detected by coronary CTA even in patients receiving statin therapy.

Therefore, we hypothesized that serum MDA-LDL levels in patients with statin therapy is involved in the prevalence of HRP by coronary CTA. The aim of this study was to clarify the association between serum MDA-LDL levels and the prevalence of HRP, which increases the likelihood of acute coronary events in patients with suspected stable coronary artery disease receiving statin therapy.

## 2. Materials and Methods

### 2.1. Study Population and Risk Assessment

**Figure 1:**
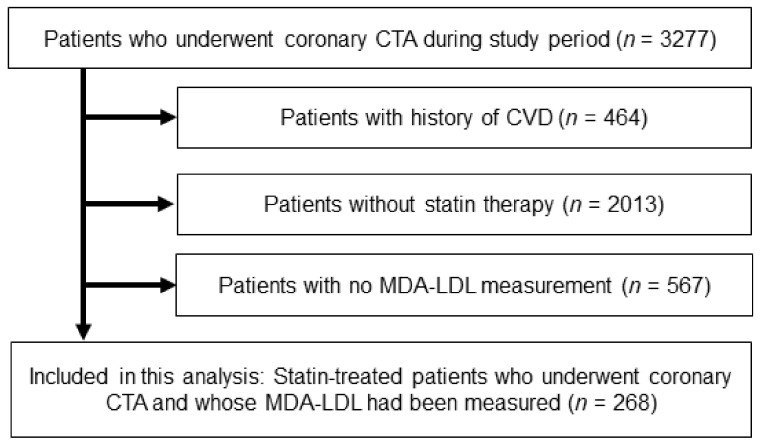
Study flowchart. CTA, computed tomography angiography; CVD, cardiovascular disease; MDA-LDL, malondialdehyde-modified low-density lipoprotein.

This single-center retrospective study included 268 outpatients who underwent coronary CTA from August 2011 to December 2018 at Okayama University Hospital. A flow diagram of this study is shown in Figure 1. Participants had no history of coronary artery disease but had been taking statins.

Hypertension was defined as systolic blood pressure (BP) ≥140 mmHg or diastolic BP ≥90 mmHg, and/or the use of antihypertensive medication. Diabetes mellitus was defined as a fasting blood glucose concentration of ≥126 mg/dL or postprandial blood glucose concentration of ≥200 mg/dL, and/or the use of insulin or oral hypoglycemic medication. medication. Smoking was defined as a self-reported history of current smoking. This study was conducted according to the principles of the Declaration of Helsinki and approved by the ethics committees of Okayama University Graduate School of Medicine, Dentistry and Pharmaceutical Sciences. All patients enrolled in the study provided written informed consent.

### 2.2. Blood Sampling and the Measurement of MDA-LDL

Blood samples were collected from the antecubital vein after fasting overnight on the day of coronary CTA. MDA-LDL levels were measured using an enzyme-linked immunosorbent assay kit (Sekisui Medical Co., Tokyo, Japan), as described previously [12].

### 2.3. Acquisition and Analyses of Coronary CTA Image

CT images were acquired using a Somatom Definition Flash scanner (Siemens Medical Solutions, Munich, Germany) as described previously [13]. Coronary artery plaques were evaluated on axial and curved multiplanar reformatted images using commercially available cardiac reconstruction software (Virtual Place, Raijin; AZE Inc., Tokyo, Japan). Interpretation of coronary CTA was evaluated by two experienced cardiologists. Significant coronary artery stenosis was defined as a luminal narrowing of >50%. Coronary plaque was defined as a structure >1 mm^2^ located within the vessel wall, and plaque density was calculated for all lesions [14]. Plaques with a CT attenuation number <50 Hounsfield units were defined as low-density plaques. Positive remodeling, which indicates an enlarged vessel to compensate for atherosclerotic change, was assessed visually on multiplanar reformatted images that were reconstructed in long-axis and short-axis views of the vessel. Positive remodeling was defined as a threshold of 1.1 for the maximal diameter of the vessel. Spotty calcification was defined as a calcium burden length <1.5 times the vessel diameter and a width of less than two-thirds of the vessel diameter. CT-verified high-risk plaques were defined when two or more plaque characteristics, including positive remodeling, low-density plaques, and spotty calcification, were present [14].

### 2.4. Outcome Data

Follow-up clinical information was obtained from a review of the medical records or telephone interviews by attending physicians. Cardiovascular events were defined as a composite of cardiac death and acute coronary syndrome. Cardiac death was defined as death from myocardial infarction, cardiogenic shock, cardiac failure, or ventricular arrhythmias. Acute coronary syndrome included myocardial infarction and unstable angina. Non-fatal myocardial infarction was defined using the criteria of typical acute chest pain and persistent ST-segment elevation or positive cardiac enzymes. Unstable angina pectoris was defined as typical acute chest pain with negative cardiac enzymes if coronary artery disease could not be excluded as the cause of symptoms in accordance with current guidelines [15].

### 2.5. Statistical Analysis

Continuous variables were expressed as mean ± standard deviation or median with interquartile range. Dichotomous variables were expressed as numbers and percentages. Differences in continuous variables between the two groups were analyzed using Student’s *t*-test and the Mann–Whitney U-test, as appropriate. Categorical data were compared by χ^2^ analysis and Fisher’s test, as appropriate. Patients were classified into two groups based on the median serum MDA-LDL level (93 U/L): high MDA-LDL group (≥93 U/L, *n* = 139) and low MDA-LDL group (<93 U/L, *n* = 129), or LDL-C level (104 mg/dL): high LDL-C group (≥104 mg/dL, *n* = 136) and low MDA-LDL group (<104 mg/dL, *n* = 132). Associations between serum MDA-LDL and each variable were assessed using Pearson’s correlation coefficient. Univariate and multivariate logistic regression analyses were performed to evaluate the association between serum MDA-LDL levels and CTA-verified HRP. A receiver operating characteristic (ROC) curve was generated to evaluated diagnostic value to predict the presence of HRP. The increased discriminative value after the addition of serum MDA-LDL levels to the baseline model in predicting the presence of HRP was assessed by the global chi-square test and ROC curve analysis. ROC curves were built based on a logistic regression model, and the Delong test was used to compare the C-statistics. The net reclassification improvement and integrated discrimination improvement were also calculated. The baseline model consisted of established clinical risk factors with *p* < 0.05 by univariate logistic regression analysis. Cumulative survival estimates were calculated using the Kaplan–Meier method and compared with the log-rank test. A Cox proportional hazard model was used to identify whether serum MDA-LDL was associated with cardiovascular events. All reported *p*-values were two-sided, and *p* < 0.05 was considered statistically significant. Statistical analyses were performed using SPSS statistical software (Version 24; IBM Corp., Armonk, NY, USA).

## 3. Results

### 3.1. Patient Characteristics

The baseline characteristics of the patients are summarized in Table 1. The mean age was 67 years, and 58% of the patients were men. A share of 76% of patients had hypertension and 46% had diabetes mellitus. Age, sex, prevalence of hypertension, diabetes mellitus, current smoking status, medications, renal function, hemoglobin A1c, and high-sensitivity CRP did not differ between the high and low MDA-LDL groups. Patients with high MDA-LDL had greater body mass index (*p* = 0.016), and higher levels of LDL-C (*p* < 0.001) and triglyceride (*p* < 0.001), than those with low MDA-LDL. Patients with high MDA-LDL had greater body mass index (*p* = 0.016), and higher levels of LDL-C (*p* < 0.001) and triglyceride (*p* < 0.001), and a lower proportion of patients achieved LDL-C <70 mg/dL, than those with low MDA-LDL.

In addition, simple correlation coefficients for the association between serum MDA-LDL and other lipid variables were analyzed. Serum MDA-LDL levels were significantly positively associated with total cholesterol (r = 0.52, *p* < 0.001), LDL-C (r = 0.60, *p* < 0.001), and log-transformed triglyceride (r = 0.34, *p* < 0.001), and were significantly inversely associated with high-density lipoprotein cholesterol (HDL) (r = −0.14, *p* = 0.025).

### 3.2. MDA-LDL and Coronary CTA Findings

**Figure 2:**
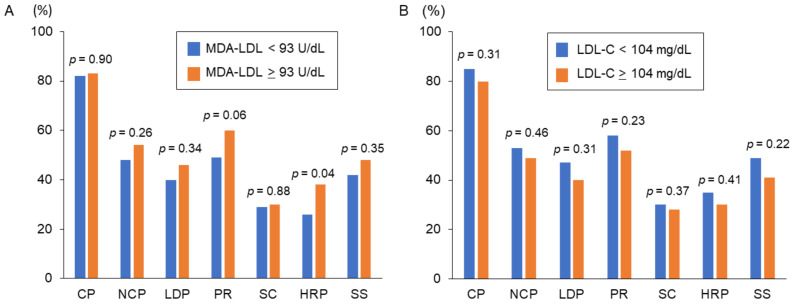
The prevalence of coronary plaque characteristics according to MDA-LDL and LDL-C. (**A**) Patients were divided into two groups based on the median value of MDA-LDL. (**B**) Patients were divided into two groups based on the median value of LDL-C. MDA-LDL, malondialdehyde-modified low-density lipoprotein; LDL-C, low-density lipoprotein cholesterol; CP, calcified plaque; NCP, non-calcified plaque; LDP, low density plaque; PR, positive remodeling; SC, spotty calcification; HRP, high-risk plaque: SS, significant stenosis.

Among all patients, the prevalence of HRP and significant stenosis presented in 87 patients (32%) and 120 patients (45%), respectively. As shown in Figure 2A, the prevalence of HRP in the high MDA-LDL group was significantly greater than that in the low MDA-LDL group (*p* = 0.040). However, no differences in the prevalence of calcified plaques (*p* = 0.904), non-calcified plaques (*p* = 0.267), low-density plaque (*p* = 0.344), positive remodeling (*p* = 0.057), spotty calcification (*p* = 0.883), and significant stenosis (*p* = 0.355) were found between the high and low MDA-LDL groups. Figure 2B shows no differences in the prevalence of HRP (*p* = 0.4111) and significant stenosis (*p* = 0.229), or other plaque features, between the high and low LDL-C groups.

As shown in Table 2, patients with HRP had a higher prevalence of male gender (*p* < 0.001), hypertension (*p* = 0.031), and diabetes mellitus (*p* = 0.028) compared with patients without HRP. Patients with HRP had lower HDL-C levels (*p* = 0.014) and serum MDA-LDL levels (*p* = 0.011), whereas LDL-C levels did not differ between patients with and without HRP. Although patients with significant stenosis had higher prevalence of male gender (*p* < 0.001) and diabetes mellitus (*p* = 0.039) compared with patients without significant stenosis, serum levels of LDL-C and MDA-LDL did not differ between patients with and without significant stenosis.

As shown in Table 3, in univariate logistic regression analysis, male sex, hypertension, diabetes mellitus, HDL, and MDA-LDL, but not LDL-C, were significantly associated with the presence of HRP. In multivariate logistic regression analysis, MDA-LDL was independently associated with the presence of HRP with an odds ratio of 1.883 (95% confidence interval 1.082–3.279, *p* = 0.025). When serum MDA-LDL levels were added to the baseline model, global chi-square scores significantly increased from 24.0 to 31.6 (*p* = 0.006). The net reclassification improvement and integrated discrimination improvement were significantly improved by 0.282 (*p* = 0.029) and 0.030 (*p* = 0.008), respectively. However, the increase in the C-statistic was not significant (0.68 to 0.70, *p* = 0.352).

### 3.3. Prognostic Impact of HRP and MDA-LDL for Cardiovascular Events

**Figure 3:**
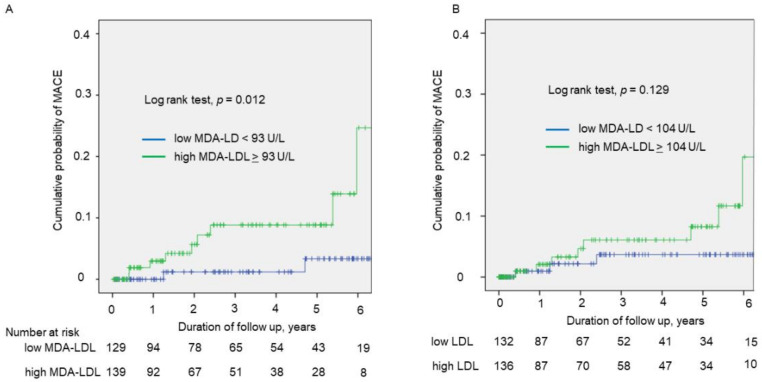
Kaplan–Meier curves of cumulative incidence of cardiovascular events. Kaplan–Meier curves (**A**) according to low or high MDA-LDL, and (**B**) according to low or high LDL-C. MDA-LDL, malondialdehyde-modified low-density lipoprotein; LDL-C, low-density lipoprotein cholesterol.

During a median follow-up period of 2.5 years, 11 cardiovascular events (two cardiac deaths, nine acute coronary syndrome) occurred. Kaplan–Meier curves showed that the high MDA-LDL group had more cardiovascular events than the low MDA-LDL group (*p* = 0.012, log-rank test) (Figure 3A), whereas no significant difference in the incidence of cardiovascular events was observed between patients with the high and low LDL-C groups (Figure 3B). Cox univariate regression analysis showed that high MDA-LDL (HR 5.717, 95%CI 1.225–26.670, *p* = 0.027), but not high LDL-C (hazard ratio 2.697, 95% confidence interval 0.715–10.183, *p* = 0.143), was significantly associated with cardiovascular events. In age and sex adjusted Cox multivariate regression analysis, high MDA-LDL was independently associated with cardiovascular events (hazard ratio 5.865, 95% confidence interval 1.250–27.512, *p* = 0.025).

## 4. Discussion

This study demonstrated that serum MDA-LDL, but not LDL-C, was significantly associated with the presence of HRP and had a moderate incremental value to predict HRP over traditional risk factors in statin-treated patients with suspected coronary artery disease. In addition, high MDA-LDL was shown to be a possible predictor for cardiovascular events defined as a composite of cardiac death and ACS in statin-treated patients.

Although increased oxidized LDL, including serum MDA-LDL, is known to be a predictor for atherosclerotic cardiovascular diseases [16], the clinical impact of serum MDA-LDL in patients receiving statin therapy has not been clarified. High triglyceride levels and low HDL levels have been known as risk markers in patients treated with lipid-lowering therapy [17]. These lipid abnormalities are associated with an increase in small dense LDL [18], which are more susceptible to oxidative modification compared with LDL. Even after considering triglyceride and HDL-C, this study clearly demonstrated that serum MDA-LDL is a relevant biomarker for the presence of HRP in statin-treated patients.

Oxidized LDL plays a critical role in plaque vulnerability. Oxidized LDL through lectin-like oxidized LDL receptor-1, which is the major receptor for oxidized LDL in endothelial cells, contributes to increased matrix metalloproteinase activity [19]. In addition, elevated concentrations of oxidized LDL induce apoptosis in vascular smooth muscle cells [20]. Enhanced matrix metalloproteinase production and apoptosis of vascular smooth muscle cells contribute to plaque instability [21]. Several previous clinical studies reported that serum MDA-LDL levels have been associated with thin-cap fibroatheroma identified by frequency-domain optical coherence tomography or with tissue characteristics evaluated by integrated backscatter intravascular ultrasound, both of which are considered to be indicators of plaque vulnerability [5,22]. However, these studies only included patients with obstructive coronary artery disease. In addition, previous studies have reported an association between serum MDA-LDL and plaque vulnerability in lesions targeted for revascularization.

Our findings suggest that serum MDA-LDL is an independent factor for cardiovascular events such as cardiac death and acute coronary syndrome. Similar to our results, a recent clinical study demonstrated that small dense LDL, which is easily oxidized to MDA-LDL, was strongly associated with myocardial infarction [23]. In fact, oxidized LDL is also reported to be involved in platelet activation [24]. The significant association between MDA-LDL and HRP shown in this study may partly explain the role of MDA-LDL as a predictor of cardiovascular events; nevertheless, further studies are required to clarify whether there is a causal relationship.

Statin therapies have been shown to decrease not only LDL but also serum MDA-LDL [25]. In fact, the current study demonstrated that LDL-C had a significant correlation with serum MDA-LDL, and the proportion of patients achieving LDL-C < 70 mg/dL in the low MDA-LDL group was significantly greater than that in the high MDA-LDL group. In addition, inflammation has been considered as a residual risk in statin-treated patients, and a previous study reported that oxidized LDL-induced interleukins (IL)-1β secretion promotes foam cell formation [26]. Recently, a clinical trial showed that antibody therapy against IL-1β significantly reduced cardiovascular events without lowering lipid or blood pressure [27]. Further studies are needed to investigate whether additional anti-inflammatory treatment in patients with high MDA-LDL will reduce cardiovascular events.

Our study has some limitations that need to be addressed. First, this was a single-center study and the number of patients was relatively small. Patient selection may have been biased and a prospective study would be preferable. Second, we included only Japanese patients with suspected stable coronary artery diseasse; the results cannot be applied to other ethnic groups and the general population. Third, the follow-up study was relatively short and a small number of cardiovascular events were documented. To determine the impact of MDA-LDL on cardiovascular death and acute coronary syndrome in statin-treated patients, a large long-term study is warranted.

## 5. Conclusions

Our study demonstrated that serum MDA-LDL was significantly associated with HRP and had an incremental value to predict HRP over traditional risk factors in statin-treated patients with suspected stable coronary artery disease. Our results suggest the measurement of serum MDA-LDL is useful in identifying patients likely to have HRP who are at high risk. Further studies with larger sample size are needed to prove the association between serum MDA-LDL and cardiovascular events in statin-treated patients.

## Figures and Tables

**Table 1:** Baseline patient demographic and clinical characteristics according to high or low levels of MDA-LDL.

	All Patients	High MDA-LDL (≥93 U/L)	Low MDA-LDL (<93 U/L)	*p*-Value
*n*	268	139	129	
Age, year	67 ± 11	67 ± 11	68 ± 11	0.521
Male sex	154 (58)	76 (55)	78 (61)	0.338
Body mass index, kg/m^2^	24.6 ± 4.1	25.2 ± 3.9	24.0 ± 4.2	0.016
Hypertension	203 (76)	109 (78)	94 (73)	0.290
Diabetes mellitus	122 (46)	59 (42)	63 (49)	0.294
Current Smoker	70 (26)	36 (26)	34 (26)	0.932
Medications				
Beta blockers	71 (27)	37 (27)	34 (26)	0.933
CCBs	122 (46)	70 (50)	52 (40)	0.099
ACE-Is or ARBs	135 (50)	73 (53)	62 (48)	0.466
Oral antihyperglycemic drugs	86 (32)	45 (32)	41 (32)	0.918
Ezetimibe	15 (6)	10 (7)	5 (4)	0.293
Statin intensity, low/moderate *	160 (60)/108 (40)	76 (55)/63 (45)	84 (65)/45 (35)	0.082
Statin type, dosage				
Atorvastatin, 5 mg/10 mg	18/49	5/26	13/23	
Fluvastatin, 20 mg/40 mg	4/1	3/1	1/0	
Pitavastatin, 1 mg/2 mg/4 mg	14/28/4	6/13/3	8/15/1	
Pravastatin, 5 mg/10 mg	9/36	5/21	4/15	
Rosuvastatin, 2.5 mg/5 mg/10 mg	68/24/2	30/19/2	38/5/0	
Simvastatin, 5 mg/10 mg	8/3	4/1	4/2	
Laboratory findings				
Creatinine, mg/dL	0.89 ± 0.78	0.85 ± 0.64	0.93 ± 0.92	0.397
eGFR, mL/min/1.73 m^2^	68 ± 18	68 ± 17	69 ± 19	0.665
Total cholesterol, mg/dL	186 ± 40	203 ± 40	168 ± 31	<0.001
LDL cholesterol, mg/dL	107 ± 32	122 ± 33	90 ± 22	<0.001
HDL cholesterol, mg/dL	58 ± 17	57 ± 17	60 ± 17	0.080
Triglyceride, mg/dL	121 (88, 171)	150 (104, 198)	101 (76, 130)	<0.001
MDA-LDL, U/L	96 ± 35	122 ± 28	69 ± 15	<0.001
HbA1c, %	6.6 ± 1.3	6.6 ± 1.4	6.6 ± 1.3	0.968
hsCRP, mg/dL	0.08 (0.05, 0.18)	0.08 (0.04, 0.16)	0.08 (0.05, 0.20)	0.608
Patients achieving LDLcholesterol <70 mg/dL	24 (9)	1 (1)	23 (18)	<0.001

Data are presented as mean ± standard deviation, number (%), or median (25th, 75th percentile). MDA-LDL, malondialdehyde low-density lipoprotein cholesterol; CCBs, calcium channel blockers; ACE-Is, angiotensin-converting enzyme inhibitors; ARBs, angiotensin receptor blockers; eGFR, estimated glomerular filtration rate; LDL, low-density lipoprotein; HDL, high-density lipoprotein; HbA1c, glycated hemoglobin A1c; hsCRP, high-sensitivity C-reactive protein. * No patients received high-intensity statins.

**Table 2:** Comparison of patient characteristics according to coronary CTA findings.

Variables	High-Risk Plaque			Significant Stenosis		
	Present	Absent	*p* Value	Present	Absent	*p* Value
*n*	87	181		119	148	
Age, year	68 ± 10	67 ± 12	0.747	68 ± 11	67 ± 12	0.489
Male sex	64 (74)	90 (50)	<0.001	84 (70)	70 (47)	<0.001
Body mass index, kg/m^2^	24.7 ± 3.4	24.5 ± 4.4	0.718	24.4 ± 4.1	24.7 ± 4.1	0.450
Hypertension	73 (84)	130 (76)	0.031	93 (78)	110 (74)	0.546
Diabetes mellitus	48 (55)	74 (41)	0.028	63 (53)	59 (40)	0.039
Current smoker	29 (33)	41 (23)	0.062	32 (27)	38 (25)	0.854
Oral antihyperglycemic drugs	32 (37)	54 (30)	0.254	42 (35)	44 (30)	0.333
Creatinine, mg/dL	1.00 ± 0.99	0.83 ± 0.65	0.087	0.91 ± 0.71	0.88 ± 0.83	0.066
Total cholesterol, mg/dL	186 ± 45	187 ± 38	0.834	183 ± 41	188 ± 38	0.300
LDL cholesterol, mg/dL	107 ± 37	107 ± 30	0.867	106 ± 34	107 ± 31	0.728
HDL cholesterol, mg/dL	55 ± 16	60 ± 17	0.014	58 ± 18	58 ± 16	0.905
Triglyceride, mg/dL	130 (90, 182)	117 (85, 170)	0.206	118 (86, 158)	122 (89, 183)	0.155
MDA-LDL, U/L	105 ± 40	92 ± 32	0.011	96 ± 33	96 ± 36	0.979
HbA1c, %	6.7 ± 1.3	6.5 ± 1.4	0.347	6.6 ± 1.3	6.5 ± 1.4	0.122
hsCRP, mg/dL	0.08 (0.05, 0.16)	0.08 (0.04, 0.19)	0.907	0.09 (0.05, 0.200)	0.07 (0.040, 0.160)	0.132

**Table 3:** Univariate and multivariate predictors of the presence of HRP.

	Univariate		Multivariate	
	Odds Ratio (95%CI)	*p* Value	Odds Ratio (95%CI)	*p* Value
Age, per 1 year	1.004 (0.981–1.028)	0.746		
Male	2.814 (1.609–4.918)	<0.001	2.749 (1.502–1.502)	0.001
Hypertension	2.046 (1.060–3.947)	0.033	2.027 (1.022–4.019)	0.049
Diabetes Mellitus	1.780 (1.062–2.982)	0.029	1.630 (0.941–2.824)	0.081
Current smoker	1.707 (0.970–3.006)	0.064		
HDL cholesterol, per 1 mg/dL	0.980 (0.964–0.996)	0.015	0.994 (0.977–1.012)	0.531
LDL cholesterol, >104 mg/dL	0.807 (0.483–1.347)	0.412		
Triglyceride *, per 1 index	1.454 (0.854–2.475)	0.168		
MDA-LDL, >93 U/L	1.722 (1.024–2.897)	0.041	1.883 (1.082–3.279)	0.025
hsCRP *, per 1 index	0.983 (0.793–1.219)	0.878		

HRP, high-risk plaques; LDL, low-density lipoprotein; HDL-C, high-density lipoprotein; MDA-LDL, malondialdehyde low-density lipoprotein cholesterol; hsCRP, high sensitivity C-reactive protein. * Triglyceride and hsCRP were logarithm-transformed.

## Data Availability

The data presented in this study are available on request from the corresponding author. The data are not publicly available due to privacy.
